# Supplementary material for: Factors and Processes Facilitating Recovery from Coercion in Mental Health Services—A Meta-Ethnography
Source: Healthcare (Basel). 2024 Mar 11;12(6):628. doi: 10.3390/healthcare12060628 (PMC10970616; doi:10.3390/healthcare12060628)
Supplement: Supplementary file 1 [file healthcare-12-00628-s001.zip › healthcare-2877259-supplementary.pdf]

Supplementary Material: S1 Quality assessment of study articles using the Critical Appraisal Skills Programme (CASP)

Quality assessment of study articles using the Critical Appraisal Skills Programme (CASP)												
Items of CASP												
	Section:	Are the results of the study valid? (Section A)							What are the results? (Section B)			Will the results help locally? (Section C)
	Paper for appraisal	1 Was there a clear statement of the aims of the research?	2 Is a qualitative methodology appropriate?	Is it worth continuing?	3 Was the research design appropriate to address the aims of the research?	4 Was the recruitment strategy appropriate to the aims of the research?	5 Was the data collected in a way that addressed the research issue?	6 Has the relationship between researcher and participants been adequately considered?	7 Have ethical issues been taken into consideration?	8 Was the data analysis sufficiently rigorous?	9 Is there a clear statement of findings?	10 How valuable is the research?
1	Andreasson & Skärsäter, 2012	Y	Y	Y	Y	Y	Y	N	Y	Y	Y	Y
2	Banks, et al. 2016	Y	Y	Y	Y	Y	Y	Y	Y	Y	Y	Y
3	Bonner, et al. 2002	Y	Y	Y	Y	Y	Y	Y	Y	Y	Y	Y
4	Canvin, et al. 2002	Y	Y	Y	Y	Y	Y	Y	Y	Y	Y	Y
5	Chambers, et al. 2014	Y	Y	Y	Y	Y	Y	Y	Y	Y	Y	Y
6	Chien, et al. 2005	Y	Y	Y	Y	Y	Y	CT	Y	Y	Y	Y
7	Ezeobele, et al. 2014 seclusion	Y	Y	Y	Y	Y	Y	Y	Y	Y	Y	Y
8	Fletcher, et al. 2019	Y	Y	Y	Y	Y	Y	CT	Y	Y	Y	Y
9	Gault, et al. 2013	Y	Y	Y	Y	Y	Y	Y	Y	Y	Y	Y
10	Hammervold, et al. 2020	Y	Y	Y	Y	Y	Y	Y	Y	Y	Y	Y
11	Hoekstra, et al. 2004	Y	Y	Y	Y	Y	Y	Y	Y	Y	Y	Y
12	Hughes, et al. 2009	Y	Y	Y	Y	Y	Y	N	Y	Y	Y	Y
13	Kontio, et al. 2012	Y	Y	Y	Y	Y	Y	Y	Y	Y	Y	Y

14	Lanthén, et al. 2015	Y	Y	Y	Y	Y	Y	Y	Y	Y	Y	Y
15	Ling, et al. 2015	Y	Y	Y	Y	Y	Y	Y	Y	Y	Y	Y
16	McGuinness, et al. 2013	Y	Y	Y	Y	Y	Y	Y	Y	Y	Y	Y
17	McGuinness, et al. 2018	Y	Y	Y	Y	Y	Y	Y	Y	Y	Y	Y
18	Murphy, et al. 2017	Y	Y	Y	Y	Y	Y	N	Y	Y	Y	Y
19	Olofsson & Jacobsson, 2001	Y	Y	Y	Y	Y	Y	Y	Y	Y	Y	Y
20	Pridham, et al. 2018	Y	Y	Y	Y	Y	Y	Y	Y	Y	Y	Y
21	Stroud, et al. 2015	Y	Y	Y	Y	Y	Y	Y	Y	Y	Y	Y
22	Wyder, et al. 2015	Y	Y	Y	Y	Y	Y	Y	Y	Y	Y	Y
23	Wyder, et al. 2016	Y	Y	Y	Y	Y	Y	Y	Y	Y	Y	Y

*Note*s. Y: Yes, CT: Can’t Tell, N: No.
Source: Critical Appraisal Skills Programme (2018). CASP (Qualitative) Checklist. [online] Available at: https://casp-uk.b-cdn.net/wp-content/uploads/2018/03/CASP-Qualitative-Checklist-2018_fillable_form.pdf. Accessed: 05.09.2022.
